# Congenital Dyserythropoietic Anemia Type III Associated With a Novel KIF23 Variant (c.2132A>G; p.Gln711Arg): A Case Report

**DOI:** 10.1002/ccr3.70875

**Published:** 2025-09-07

**Authors:** Ahmad Hamammdi, Kareem Sultan Tamimi, Adam Qabaha, Omar Bsharat, Kareem Ibraheem, Ahmad Batran

**Affiliations:** ^1^ Faculty of Medicine Palestine Polytechnic University Hebron Palestine; ^2^ Palestinian Clinical Research Center Bethlehem Palestine

**Keywords:** anemia, dyserythropoietic, genetic testing, hematology, KIF23 mutation, kinesins

## Abstract

Congenital dyserythropoietic anemia type III (CDA III) is an extremely rare inherited disorder characterized by ineffective erythropoiesis, multinucleated erythroblasts in the bone marrow, and variable clinical gravity. We report the case of a 6‐year‐old boy, presenting with abdominal distension, failure to thrive, dark urine, intermittent itching, and recurrent infections. Physical examination revealed pallor, hepatomegaly, and splenomegaly. Laboratory investigations showed mild normocytic anemia, high liver enzymes, and erythroid hyperplasia. The sequencing of the whole exoma identified a variant of uncertain meaning in the KIF23 gene, which is implicated in cytokinesis and attached to the pathogenesis of CDA III. The patient remains clinically stable in support management, without the current need for transfusion. This case highlights the importance of advanced genetic tests in the diagnosis of rare hematological conditions and expands the potential spectrum of mutations associated with CDA III. Early recognition and long‐term monitoring are essential for guiding management and monitoring complications such as iron overload and splenomegaly.

**Summary**

- This case highlights that Congenital Dyserythropoietic Anemia Type III (CDA III), while rare, should be considered in children presenting with failure to thrive, hepatosplenomegaly, and mild anemia.
- Despite often lacking significant transfusion dependency, genetic testing, such as whole exome sequencing (WES), is crucial for definitive diagnosis, especially when classic bone marrow findings are absent or inconclusive.
- Management is primarily supportive, focusing on monitoring and addressing potential complications like splenomegaly and iron overload.

## 1 Introduction

Congenital dyserythropoietic anemias encompass a wide range of inherited disorders characterized by hemolysis with ineffective erythropoiesis [1]. The resultant anemia is classified according to morphological bone marrow, clinical, and genetic features into five types [2]. These disorders are typically diagnosed in childhood or early adulthood, although milder cases may escape detection until later in life. Congenital dyserythropoietic anemia type III (CDA III) is a very rare subtype characterized by the presence of giant erythroblasts in the bone marrow with erythrocytosis [3]. The molecular basis of CDA III is linked to mutations in the KIF23 gene. This gene encodes mitotic kinesin, which is responsible for cytokinesis, resulting in cell division impairment and the formation of multinucleated cells [3, 4]. Clinical features range from mild asymptomatic patients to severe transfusion‐dependent patients, and in some cases, splenomegaly can occur with skeletal anomaly [3, 5]. Diagnosis is based on three things, which are a genetic study to confirm (KIF23), a hematological study, and bone marrow aspiration. Management is supportive, including blood transfusions for severe anemia and splenectomy for symptomatic splenomegaly [5]; however, novel therapeutic approaches targeting the underlying molecular defect are under investigation.

## 2 Case History/Examination

A 6‐year‐old male presented with abdominal distension and a history of failure to thrive. Additional symptoms included dark urine, intermittent pruritus, and recurrent infections. The family history was significant for renal stones, β‐thalassemia, and a cousin diagnosed with hypoplastic portal vein and liver cirrhosis. On general examination, the child was alert, active, and cooperative with no dysmorphic features or lymphadenopathy. Pallor was noted. Growth measurements showed a weight of 15.4 kg and a height of 103 cm, both below the 5th percentile for age, consistent with failure to thrive. Head circumference was 48.5 cm, placing it between the 3rd and 5th percentiles (lower end of normal).

The abdomen was distended and symmetrical with normoactive bowel sounds. Palpation revealed a soft abdomen with no guarding or rebound tenderness. The liver was palpable 6 cm below the right costal margin, hard, with a span of approximately 14 cm, indicating hepatomegaly. The spleen was palpable 8 cm below the left costal margin, consistent with splenomegaly. No other abdominal masses or hernias were found. Percussion was normal, with no evidence of shifting dullness.

Fundoscopic and central nervous system examinations were unremarkable, with normal muscle power and tone, and no focal neurological deficits. The remainder of the examination was unremarkable. There was no history of NICU admission or neonatal complications.

## 3 Differential Diagnosis, Investigations and Treatment

The patient, a child presenting with failure to thrive, hepatosplenomegaly, and recurrent infections, initially prompted consideration of cystic fibrosis. However, this diagnosis was subsequently excluded by normal sweat chloride test results and the absence of CFTR gene mutations.

Initial laboratory investigations revealed mild normocytic anemia (Table 1) (Hb: 12.4 g/dL) without hyperbilirubinemia (total bilirubin: 0.4 mg/dL, direct bilirubin: 0.26 mg/dL), alongside elevated liver enzymes. A lipid profile showed cholesterol at 151 mg/dL, triglycerides at 133 mg/dL, HDL at 32.8 mg/dL, and LDL at 92 mg/dL.

**TABLE 1:** Laboratory evaluation showed mild normocytic, normochromic anemia (HGB 12.4 g/dL, HCT 37.3%) with an elevated reticulocyte count (2.52%), indicating a compensatory bone marrow response.

Test name	Result	Normal value	Unit
Red blood cell distribution width coefficient of variation	13.1	—	%
White blood cells	9.1	—	K/uL
Red blood cells (RBC)	4.43	—	M/uL
Hemoglobin (HGB)	12.4	13.5–17.5	g/dL
Hematocrit (HCT)	37.3	41–53	%
Mean cell volume (MCV)	84.2	80–100	fL
Mean cell hemoglobin (MCH)	28	25–35	pg/cell
Mean cell hemoglobin concentration (MCHC)	33.2	31–35	g/dL
Platelet count	281	—	K/uL
Reticulocytes	2.52	0.5–1.5	%
Lactate dehydrogenase (LDH)	180	45–200	U/L

*Note:* Red cell indices were normal, and LDH levels (180 U/L) were within the reference range, making significant hemolysis less likely. Platelet and white cell counts were normal.

Abdominal ultrasonography confirmed hepatomegaly (14 cm in the midclavicular line) and splenomegaly (12.5 cm), with normal echotexture of both organs and a partially distended gallbladder lacking stones. Peripheral blood smear analysis demonstrated severe erythroid hyperplasia with notable anisocytosis and poikilocytosis.

Further diagnostic inquiry, specifically whole exome sequencing (WES), revealed a novel heterozygous single nucleotide variant of uncertain significance, c.2132A>G (p.Gln711Arg), in the KIF23 gene.

The KIF23 gene encodes a kinesin protein integral to cytokinesis. While this particular variant has not been previously implicated in CDA III, its potential impact on cell division pathways suggests a plausible pathogenic role, warranting further investigation into its functional implications.

Molecular genetic analysis for the proband demonstrated an average sequencing coverage of 137.76X. Although the heterozygous state of the identified KIF23 variant was confirmed, its precise variant allele frequency (VAF) was not explicitly quantified within the report.

Currently, supportive care is being provided, and the patient remains under close follow‐up to monitor disease progression and assess the efficacy of conservative management strategies.

## 4 Conclusion and Results (Outcome and Follow‐Up)

The clinical, hematologic, and genetic findings were consistent with CDA III, although the KIF23 variant identified was classified as a variant of uncertain significance. The patient remains clinically stable on supportive management, with no need for transfusions thus far. Continued genetic counseling and monitoring are ongoing, and future follow‐up will determine whether additional interventions are necessary. The case highlights the role of advanced genetic testing in diagnosing rare inherited hematologic disorders and expands the potential mutation spectrum associated with CDA III.

## 5 Discussion

CDA‐III is the rarest subtype of dyserythropoietic anemia, usually inherited as an autosomal dominant disorder due to mutations in the KIF23 gene that codes for Mitotic Kinesin‐Like Protein 1 (MKLP1), a centralspindlin component important for cytokinesis [1, 2, 3]. Failure of cytokinesis by MKLP1 depletion leads to the occurrence of large multinucleate erythroblasts in the bone marrow [2], indicating that CDA III pathogenesis is based on a cytokinesis defect. Mutations in the RACGAP1 gene, also a centralspindlin component, were recently reported as the cause of the autosomal recessive type of CDA III, suggesting a shared pathogenesis, as both genes are part of centralspindlin [6, 7].

Clinically, patients with CDA III typically present with no or moderate anemia, normal or mildly elevated mean corpuscular volume (MCV), mild relative reticulocytopenia, jaundice, and signs of hemolysis. Unlike CDA types I and II, splenomegaly is typically absent in CDA III [4, 8, 9]. Bone marrow examination often reveals erythroid hyperplasia with giant multinucleated erythroblasts, a hallmark feature of CDA III on light microscopy [4, 5]. Our patient presented with mild anemia, pallor, dark urine, hepatosplenomegaly with significant splenomegaly, poor growth history, and no significant history of transfusion dependency, which correlates with the presentation described in the literature of CDA III.

WES confirmed a KIF23 mutation, reinforcing its diagnostic value in distinguishing CDA III from other hemolytic anemias, including hereditary spherocytosis, pyruvate kinase deficiency, or others, which can share overlapping morphologic bone marrow results [3]. This case further confirms the pathogenicity of KIF23 mutations in CDA III and emphasizes the utility of genetic testing in definitive diagnosis.

The treatment of CDA III is mainly supportive. Most cases do not require treatment. Though transfusion may be required during episodes of severe anemia, for example, pregnancy and after surgery. People with eye symptoms should have regular ophthalmological examinations. In certain cases, splenomegaly may be treated by a splenectomy, which can improve anemia but increases infection risk [10]. Iron overload is a concern in patients requiring frequent transfusions, and iron chelation is recommended to prevent end organ damage [4, 5, 10]. Bone marrow transplantation has been reported as a curative treatment, but it is generally reserved for severe cases due to the procedure's significant associated morbidity and mortality [11].

In conclusion, this case reinforces the role of KIF23 mutations in CDA III and demonstrates the importance of WES in establishing a definitive diagnosis. While treatment remains supportive, awareness of complications like splenomegaly and iron overload is essential for optimal management.

## Author Contributions

**Ahmad Hamammdi:** conceptualization, writing – original draft. **Kareem Sultan Tamimi:** conceptualization, supervision, writing – review and editing. **Adam Qabaha:** conceptualization, writing – review and editing. **Omar Bsharat:** writing – review and editing. **Kareem Ibraheem:** supervision, writing – review and editing. **Ahmad Batran:** supervision, writing – original draft.

## Consent

Written informed consent was obtained from the patient parents for the publication of this case report.

## Conflicts of Interest

The authors declare no conflicts of interest.

## Data Availability

The data used to support the findings of this study are included in the article.
